# Association of the Implementation of Child Tax Credit Advance Payments With Food Insufficiency in US Households

**DOI:** 10.1001/jamanetworkopen.2021.43296

**Published:** 2022-01-13

**Authors:** Paul R. Shafer, Katherine M. Gutiérrez, Stephanie Ettinger de Cuba, Allison Bovell-Ammon, Julia Raifman

**Affiliations:** 1 Department of Health Law, Policy, and Management, School of Public Health, Boston University, Boston, Massachusetts; 2 Department of Economics, Williams College, Williamstown, Massachusetts; 3 Children’s HealthWatch, Boston Medical Center, Boston, Massachusetts; 4 Department of Pediatrics, School of Medicine, Boston University, Boston, Massachusetts

## Abstract

**Importance:**

A key component of the American Rescue Plan Act of 2021 included an expansion of the Child Tax Credit with advance payments beginning in July 2021, a “child allowance” that was projected to dramatically reduce child poverty. Food insufficiency has increased markedly during the economic crisis spurred by the COVID-19 pandemic, with disparities among marginalized populations, and may be associated with substantial health care and social costs.

**Objective:**

To assess whether the introduction of advance payments for the Child Tax Credit in mid-July 2021 was associated with changes in food insufficiency in US households with children.

**Design, Setting, and Participants:**

This cross-sectional study used data from several phases of the Household Pulse Survey, conducted by the US Census Bureau from January 6 to August 2, 2021. The survey had 585 170 responses, representing a weighted population size of 77 165 153 households.

**Exposure:**

The first advance Child Tax Credit payment, received on July 15, 2021.

**Main Outcomes and Measures:**

Household food insufficiency.

**Results:**

The weighted sample of 585 170 respondents was mostly female (51.5%) and non-Hispanic White (62.5%), with a plurality aged 25 to 44 years (48.1%), having a 4-year degree or more (34.7%) and a 2019 household income of $75 000 to $149 999 (23.1%). In the weeks after the first advance payment of the Child Tax Credit was made (July 21 to August 2, 2021), 62.4% of households with children reported receiving it compared with 1.1% of households without children present (*P* < .001). There was a 3.7–percentage point reduction (95% CI, –0.055 to –0.019 percentage points; *P* < .001) in household food insufficiency for households with children present in the survey wave after the first advance payment of the Child Tax Credit, corresponding to a 25.9% reduction, using an event study specification. Difference-in-differences (−16.4%) and modified Poisson (−20.8%) models also yielded large estimates for reductions in household food insufficiency associated with the first advance payment of the expanded Child Tax Credit.

**Conclusions and Relevance:**

This study suggests that the Child Tax Credit advance payment increased household income and may have acted as a buffer against food insufficiency. However, its expansion and advance payment are only a temporary measure for 2021. Congress must consider whether to extend these changes or make them permanent and improve implementation to reduce barriers to receipt for low-income families.

## Key Points

**Question:**

Was the introduction of advance payments for the Child Tax Credit associated with food insufficiency in US households?

**Findings:**

In this cross-sectional study using repeated surveys of a nationally representative sample of US households, the introduction of advance payments for the Child Tax Credit was associated with a significant reduction in household food insufficiency of approximately 26%.

**Meaning:**

This study suggests that the Child Tax Credit advance payments acted as a buffer against food insufficiency among households with children.

## Teaser

This cross-sectional study uses data from the Household Pulse Survey to assess whether the introduction of advance payments for the Child Tax Credit in mid-July 2021 was associated with changes in food insufficiency in US households with children.

## Introduction

The COVID-19 pandemic caused more than 50 million people to lose work, mostly in low-income households.^1^ Food insufficiency, when households sometimes or often do not have enough to eat, rose dramatically during the economic crisis spurred by the pandemic.^2,3^ Households with children were most affected—food insufficiency in households with children peaked at 18% in December 2020 and remained at 14% through June 2021, compared with approximately 3% among all households during 2019.^4,5^ Structural racism has shaped disparities in food insufficiency, with children in Black non-Hispanic (19.2%) and Hispanic (22.0%) households experiencing food insufficiency at almost triple the rate of children in White non-Hispanic households (7.0%) as of June 2021.^3^ This indicator of material hardship is associated with harm to child development and health, higher health care costs, and nearly $170 billion annually in lost productivity, educational performance, and food aid.^6,7,8,9^

The American Rescue Plan Act (ARPA), a $1.9 trillion economic stimulus package passed in March 2021, contained several investments designed to reduce economic precarity.^10^ A key component of ARPA was a 1-year expansion of the Child Tax Credit (CTC), with 3 major reforms: (1) eligibility for the full credit amount including families with low or no income, (2) increased credit from $2000 per qualifying child to $3000 for those aged 6 to 17 years (previously only eligible up to age 16 years) and $3600 for those aged 5 years or younger, and (3) advance payments made on a monthly basis.^11,12^ These changes were recommended by the National Academies of Science, Engineering, and Medicine to reduce child poverty.^11^ This first reform to the CTC noted above has important equity implications, as Black, Hispanic, and Native American families often earned too little to receive the full benefit; however, the tax filing requirement for automatic receipt of the advance payments may still differentially disadvantage Black, Hispanic, and Native American families, calling into question how much this policy change can help narrow racial and ethnic disparities in reality without adjustments to its implementation.

Multiple studies before the COVID-19 pandemic demonstrated the short-term and long-term associations of participation in public assistance programs and receipt of refundable tax credits with food security and family economic stability.^13,14,15,16,17^ Prior research during the COVID-19 pandemic demonstrated that economic supports, such as enhanced unemployment benefits and a higher minimum wage, were associated with reduced food insufficiency.^18,19^ Our objective was to assess whether the first CTC advance payment on July 15, 2021, was associated with changes in household food insufficiency, building on descriptive findings recently released.^20^

## Methods

Our study uses data from the Household Pulse Survey, a recurring nationally representative, cross-sectional online survey of US households conducted by the US Census Bureau, for January 6 to August 2, 2021.^21^ This study period includes data from Phases 3 (January 6 to March 29, 2021), 3.1 (April 14 to July 5, 2021), and 3.2 (July 21 to August 2, 2021). Only 1 individual per sampled household is invited to respond, providing responses regarding both themselves and their household. We limited the sample to respondents younger than 65 years (n = 667 836) to focus on working-age adults with children in their household. The outcome was household food insufficiency, based on the survey item “Getting enough food can also be a problem for some people. In the last 7 days, which of these statements best describes the food eaten in your household?” We coded our outcome as 1 if respondents reported sometimes or often “not [having] enough food to eat” in the last 7 days, and 0 otherwise (“enough of the kinds of food [I/we] wanted to eat,” and “enough, but not always the kinds of food [I/we] wanted to eat”). This coding is the same as others using the Household Pulse Survey data in recent studies during the COVID-19 pandemic.^18,22,23^ Although closely related, food insufficiency is not the same as food insecurity, a more expansive construct based on an 18-item scale developed by the US Department of Agriculture. Food insufficiency is a narrower construct, focusing on quantity and possibly quality of food intake, representing a single measure of this scale, with both the single item and scale having been validated as household-level outcomes.^24^ This study was determined not to be human subjects research by the Boston University Medical Campus Institutional Review Board because deidentified public use data were used. Our study meets the Strengthening the Reporting of Observational Studies in Epidemiology (STROBE) reporting guideline for cross-sectional studies.

We used an event study specification to estimate the association between household food insufficiency and the exposure of being in a household with children present after the introduction of the CTC advance payments. This approach takes advantage of a natural experiment, the introduction of advance payments of the CTC in July 2021, which we expected to have a differential association with households with children present, as only those households were eligible to receive the advance payments (completely phases out to $0 at $240 000 of household income for single filers and $440 000 for married individuals filing jointly). An event study is a more flexible version of a traditional difference-in-differences regression model, the latter of which relies on treatment and poststudy period indicators and their interaction to identify a differential association. The event study instead uses survey wave indicators and the interactions between them and the treatment group indicator (household with children present) that accounts for variability in the outcome during the study period. A crucial assumption underlying the event study and difference-in-differences regression framework is parallel trends, during both the prestudy period (observed) and counterfactual poststudy period (unobserved). On graphical (eFigure 1 in the Supplement) and statistical evaluation, there were no concerns about nonparallel trends in household food insufficiency between the treated (households with children present) and untreated (households without children present) groups. Using a naive and fully adjusted model on the prestudy period data only with the treatment group indicator, linear time trend, and their interaction, no significant difference was seen in the trends between the treated and untreated groups. Covariates included respondent sex at birth (female or male), age group (18-24, 25-44, or 45-65 years), race and ethnicity (non-Hispanic White, Hispanic, non-Hispanic Black, non-Hispanic Asian, or another race or ethnicity, reflecting all categories available in the public use files [other categories collected but not made available in public data files because of small numbers]), educational level (less than high school, high school or equivalent, some college or 2-year degree, or 4-year degree or more), household income in 2019 (<$25 000, $25 000-$34 999, $35 000-$49 999, $50 000-$74 999, $75 000-$149 999, ≥$150 000, or missing), marital status (married or not), number of adults in the household (1, 2, or ≥3), number of children in the household (0, 1, 2, or ≥3), employment for the respondent in the last 7 days (yes or no), receipt of unemployment insurance benefits by the respondent in the last 7 days (yes or no), current receipt of Supplemental Nutrition Assistance Program (SNAP) benefits by anyone in the household (yes or no), receipt of food aid (eg, food pantry) by anyone in the household in the last 7 days (yes or no), receipt of Economic Impact Payment (stimulus) by anyone in the household in the last 7 days (yes or no), health insurance coverage (uninsured, public, or private), and state fixed effects.

We also ran difference-in-differences (same exposure) and modified Poisson models (using self-reported receipt of a CTC advance payment as exposure) as alternative specifications. The modified Poisson model does not make use of the natural experiment, relying on self-reported receipt of a CTC advance payment rather than the known targeting of the policy to households with children present, and provides adjusted prevalence ratios, which we report while also estimating marginal effects to enable direct comparison with the difference-in-differences and event study results. The modified Poisson model is preferable to a logistic regression model, even when odds ratios are converted to risk ratios, as it is more robust under model misspecification.^25,26,27^ We used complete-case analysis, except for income, resulting in a sample of 585 170 responses, representing a weighted population size of 77 165 153 households (82 666 observations [12.4%] removed). We used household survey weights divided by the 13 waves in our sample and clustered SEs by state, a more conservative approach than using the balanced repeated replication weights recommended by the US Census Bureau.^22^ Two-sided *t* tests or χ^2^ tests were used to test for significant differences in characteristics between households with and households without children. *P* < .05 was considered significant. Our analysis was conducted in August 2021 in Stata/MP, version 16 (StataCorp).

## Results

**Figure 1.**
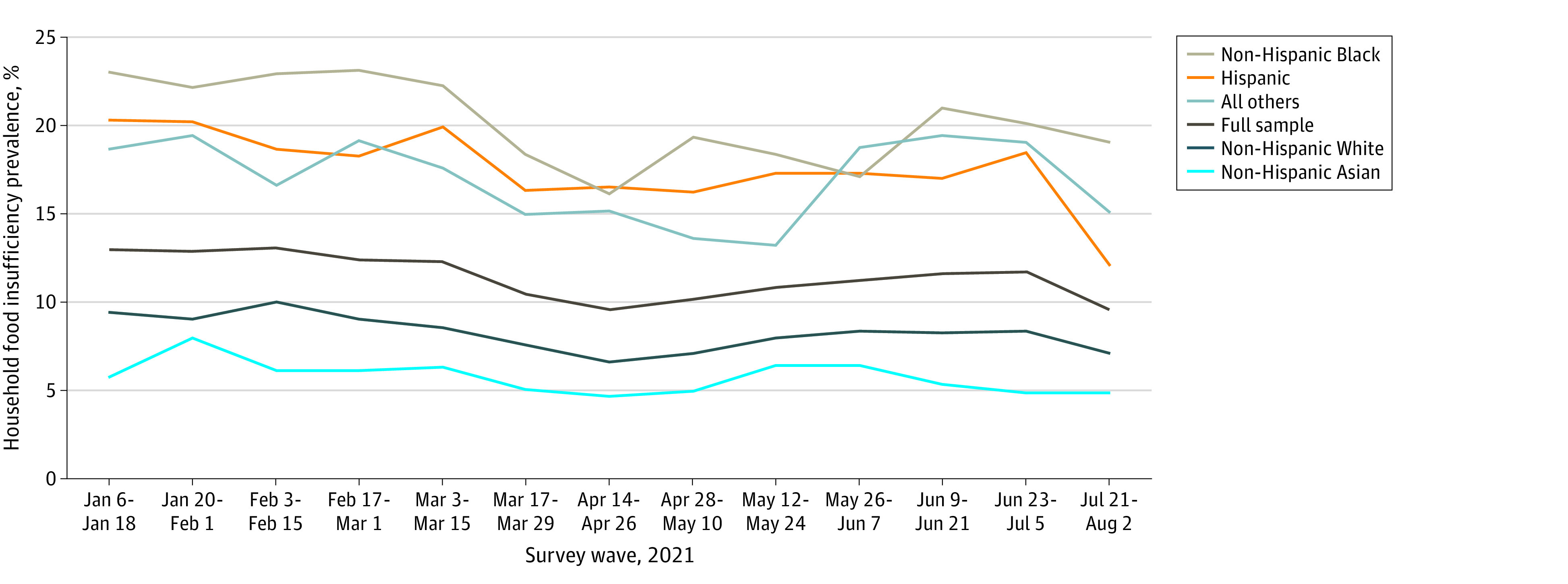
Household Food Insufficiency by Race and Ethnicity, Census Bureau Household Pulse Survey, January to August 2021 Child Tax Credit advance payments introduced July 15, 2021.

Our weighted sample of 585 170 responses was mostly female (51.5%) and non-Hispanic White (62.5%), with a plurality aged 25 to 44 years (48.1%), having a 4-year degree or more (34.7%) and a 2019 household income of $75 000 to $149 999 (23.1%). Weighted individual demographics and household socioeconomic characteristics of our analytic sample are shown in Table 1 for the full sample and stratified by presence of children in the household. In the weeks after the first CTC advance payment was made (July 21 to August 2, 2021), 62.4% of households with children reported receiving it compared with 1.1% of households without children present (*P* < .001). Unadjusted household food insufficiency decreased by 2.1 percentage points (*P* < .001), from 11.7% just before the CTC advance payment (June 23 to July 5, 2021) to 9.6% after the payment (July 21 to August 2, 2021) (Figure 1; eTable 1 in the Supplement). This decline was larger in households with children present, where household food insufficiency decreased by 4.4 percentage points (*P* < .001), from 14.3% before the CTC advance payment to 9.9% after the payment (eTable 1 in the Supplement). Non-Hispanic Asian and White individuals consistently reported the lowest rates of household food insufficiency, while non-Hispanic Black, Hispanic, and all other individuals reported rates higher than the national mean (Figure 1; eTable 1 in the Supplement). Among households with children present, unadjusted household food insufficiency declined most among Hispanic respondents (from 22.3% just before the CTC advance payment to 12.3% after the payment), while it decreased least among Black respondents (from 22.3% just before the CTC advance payment to 21.1% after the payment) (eTable 1 in the Supplement). Changes in household food insufficiency after the first CTC advance payment by state (eFigure 2 in the Supplement) varied widely, with highest prevalence concentrated in the South (eFigure 3 in the Supplement).

**Table 1.** Sample Characteristics, Census Bureau Household Pulse Survey, January to August 2021^a^

Characteristic	No. (weighted %)			*P* value
	Full sample (n = 585 170)	Households with children (n = 249 396)	Households without children (n = 335 774)	
Sex at birth				
Female	360 380 (51.5)	159 580 (55.5)	200 800 (48.4)	<.001
Male	224 790 (48.5)	89 816 (44.6)	134 974 (51.6)	
Age group, y				
18-24	19 349 (7.7)	5989 (6.3)	13 360 (8.8)	<.001
25-44	236 644 (48.1)	135 516 (60.5)	101 128 (38.4)	
45-64	329 177 (44.3)	107 891 (33.2)	221 286 (52.9)	
Race and ethnicity				
Non-Hispanic White	420 003 (62.5)	169 327 (56.3)	250 676 (67.2)	<.001
Hispanic	63 157 (16.1)	32 161 (19.9)	30 996 (13.2)	<.001
Non-Hispanic Black	45 285 (12.3)	21 187 (14.1)	24 098 (11.0)	<.001
Non-Hispanic Asian	33 243 (5.2)	15 980 (5.6)	17 263 (4.9)	<.001
Another race or ethnicity^b^	23 482 (3.8)	10 741 (4.0)	12 741 (3.7)	<.001
Educational level				
<High school	12 047 (7.0)	6864 (9.2)	5183 (5.2)	<.001
High school or equivalent	62 456 (27.5)	26 482 (27.7)	35 974 (27.3)	
Some college or 2-y degree	183 576 (30.9)	75 120 (30.2)	108 456 (31.3)	
4-y Degree or more	327 091 (34.7)	140 930 (32.9)	186 161 (36.2)	
Household income, $				
<25 000	49 755 (12.8)	17 195 (11.6)	32 560 (13.8)	<.001
25 000-34 999	37 731 (9.0)	14 599 (8.7)	23 132 (9.1)	
35 000-49 999	47 875 (9.7)	17 865 (8.9)	30 010 (10.4)	
50 000-74 999	80 296 (13.8)	30 201 (12.6)	50 095 (14.8)	
75 000-149 999	166 103 (23.1)	71 519 (22.7)	94 584 (23.3)	
≥150 000	110 904 (12.5)	53 192 (13.5)	57 712 (11.7)	
Missing	92 506 (19.2)	44 825 (22.0)	47 681 (17.0)	
Marital status				
Married	343 713 (50.9)	179 210 (63.1)	164 503 (41.4)	<.001
Not married	241 457 (49.1)	70 186 (36.9)	171 271 (58.6)	
No. of adults in household				
1	114 275 (20.4)	33 320 (14.9)	80 955 (24.6)	<.001
2	321 440 (51.9)	152 110 (57.3)	169 330 (47.8)	
≥3	149 455 (27.7)	63 966 (27.8)	85 489 (27.6)	
No. of children in household				
0	335 774 (56.3)	0	335 774 (100.0)	<.001
1	107 855 (19.0)	107 855 (43.4)	0	
2	92 139 (15.5)	92 139 (35.3)	0	
≥3	49 402 (9.3)	49 402 (21.3)	0	
Employment for respondent in the last 7 d	430 574 (69.1)	187 025 (68.7)	243 549 (69.4)	.002
Receipt of unemployment insurance benefits by respondent in the last 7 d	12 357 (2.7)	5245 (3.0)	7112 (2.5)	<.001
Current receipt of Supplemental Nutrition Assistance Program benefits by anyone in the household	49 386 (12.8)	28 809 (18.2)	20 577 (8.6)	<.001
Receipt of food aid in the last 7 d by anyone in the household	36 886 (8.0)	23 615 (11.7)	13 271 (5.1)	<.001
Receipt of Economic Impact Payment in the last 7 d by anyone in the household	37 047 (8.4)	16 710 (9.1)	20 337 (7.8)	<.001
Health insurance coverage				
Uninsured	87 554 (20.8)	37 822 (21.7)	49 732 (20.1)	<.001
Public	50 406 (10.9)	22 721 (12.6)	27 685 (9.6)	
Private	447 210 (68.3)	188 853 (65.7)	258 357 (70.3)	

^a^ Limited to working-age respondents (<65 years of age), weighted using household survey weights divided by number of waves. Demographic characteristics are specific to the respondent; receipt of public assistance and other support is either individual or household, as indicated. Two-sided *t* tests or χ^2^ tests were used to test for significant differences in characteristics between households with and without children.

^b^ Reflects all categories available in the public use files (other categories collected but not made available in public data files because of small numbers).

**Figure 2.**
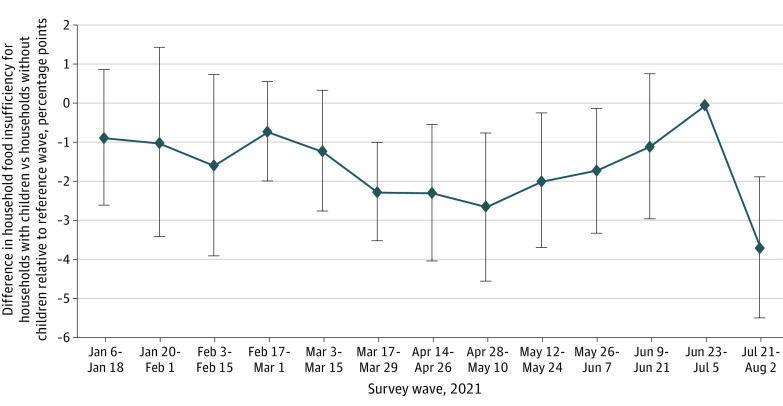
Event Study Marginal Effects on Household Food Insufficiency by Survey Wave for Households With Children Present, Census Bureau Household Pulse Survey, January to August 2021 Error bars represent 95% CIs. Reference wave is week 33 of Phase 3.1 (June 23 to July 5, 2021), comparing changes in household food insufficiency for households with children present (those targeted by the Child Tax Credit advance payments) with those without children present (not targeted by the Child Tax Credit advance payments). Child Tax Credit advance payments introduced July 15, 2021.

Our event study specification estimated a 3.7–percentage point reduction (95% CI, –0.055 to –0.019 percentage points; *P* < .001) in household food insufficiency associated with the survey wave after the first CTC advance payment (July 21 to August 2, 2021) among households with children relative to the survey wave just prior, a 25.9% relative reduction (Table 2; Figure 2). For comparison, a less flexible difference-in-differences model (eTable 2 in the Supplement) estimated a 2.2–percentage point reduction (95% CI, –0.035 to –0.010 percentage points; *P* = .001) in household food insufficiency after the first CTC advance payment among households with children compared with the prestudy period mean (January 6 to July 5, 2021), a 16.4% decline. Our modified Poisson regression model (eTable 3 in the Supplement) showed that self-reported receipt of a CTC advance payment in the last 4 weeks was associated with a 20.8% reduction (adjusted prevalence ratio, 0.792; 95% CI, 0.670-0.936; *P* = .007) in the probability of household food insufficiency relative to households that did not receive a payment, a decrease of 2.4 percentage points. Our results did not significantly change when state fixed effects were excluded from the models or state-specific time trends were included.

**Table 2.** Event Study Estimates of Association of Introduction of Child Tax Credit Advance Payments With Household Food Insufficiency^a^

Characteristic	Household food insufficiency (N = 585 170)	
	Coefficient (95% CI)	*P* value
Survey wave indicator in 2021		
January 6-18	0.015 (0.005 to 0.025)	.004
January 20 to February 1	0.011 (–0.005 to 0.028)	.18
February 3-15	0.017 (0.003 to 0.031)	.02
February 17 to March 1	0.007 (–0.005 to 0.020)	.23
March 3-15	0.009 (–0.003 to 0.020)	.13
March 17-29	–0.003 (–0.013 to 0.008)	.63
April 14-26	–0.012 (–0.023 to 0.0002)	.06
April 28 to May 10	–0.003 (–0.017 to 0.011)	.64
May 12-24	–0.001 (–0.013 to 0.012)	.90
May 26 to June 7	0.002 (–0.015 to 0.019)	.79
June 9-21	0.001 (–0.011 to 0.013)	.82
June 23 to July 5	0 [Reference]	NA
July 21 to August 2	–0.007 (–0.018 to 0.004)	.19
Presence of children in household indicator	0.045 (0.030 to 0.061)	<.001
Survey wave × presence of children in household interaction in 2021		
January 6-18	–0.008 (–0.026 to 0.009)	.32
January 20 to February 1	–0.010 (–0.034 to 0.014)	.42
February 3-15	–0.016 (–0.039 to 0.008)	.18
February 17 to March 1	–0.007 (–0.019 to 0.006)	.27
March 3-15	–0.012 (–0.027 to 0.004)	.13
March 17-29	–0.022 (–0.035 to –0.010)	.001
April 14-26	–0.023 (–0.040 to –0.005)	.01
April 28 to May 10	–0.026 (–0.045 to –0.007)	.007
May 12-24	–0.020 (–0.037 to –0.002)	.03
May 26-June 7	–0.017 (–0.033 to –0.001)	.04
June 9-21	–0.011 (–0.029 to 0.008)	.25
June 23 to July 5	0 [Reference]	NA
July 21 to August 2	–0.037 (–0.055 to –0.019)	<.001
Sex at birth		
Female	–0.006 (–0.009 to –0.003)	.001
Male	0 [Reference]	NA
Age group		
18-24 y	0 [Reference]	NA
25-44 y	0.051 (0.042 to 0.059)	<.001
45-64 y	0.027 (0.017 to 0.037)	<.001
Race and ethnicity		
Non-Hispanic White	0 [Reference]	NA
Hispanic	0.024 (0.013 to 0.035)	<.001
Non-Hispanic Black	0.053 (0.046 to 0.060)	<.001
Non-Hispanic Asian	–0.009 (–0.014 to –0.005)	<.001
Another race or ethnicity^b^	0.046 (0.036 to 0.056)	<.001
Educational level		
<High school	0 [Reference]	NA
High school or equivalent	–0.073 (–0.088 to –0.058)	<.001
Some college or 2-y degree	–0.098 (–0.113 to –0.083)	<.001
4-y Degree or more	–0.135 (–0.149 to –0.121)	<.001
Household income, $		
<25 000	0 [Reference]	NA
25 000-34 999	–0.053 (–0.062 to –0.043)	<.001
35 000-49 999	–0.090 (–0.101 to –0.078)	<.001
50 000-74 999	–0.128 (–0.139 to –0.118)	<.001
75 000-149 999	–0.150 (–0.161 to –0.139)	<.001
≥150 000	–0.147 (–0.157 to –0.137)	<.001
Missing	–0.129 (–0.139 to –0.119)	<.001
Marital status		
Married	–0.025 (–0.020 to –0.009)	<.001
Not married	0 [Reference]	NA
No. of adults in household		
1	0 [Reference]	NA
2	–0.015 (–0.020 to –0.009)	<.001
≥3	–0.005 (–0.014 to 0.004)	.26
No. of children in household		
0	0 [Reference]	NA
1	–0.014 (–0.024 to –0.005)	.004
2	–0.015 (–0.025 to –0.006)	.002
≥3	0 [Omitted]	NA
Employment for respondent in last 7 d	–0.055 (–0.060 to –0.050)	<.001
Receipt of unemployment insurance benefits by respondent in last 7 d	0.002 (–0.013 to 0.018)	.76
Current receipt of Supplemental Nutrition Assistance Program benefits by anyone in household	0.022 (0.009 to 0.035)	.001
Receipt of food aid in last 7 d by anyone in household	0.078 (0.071 to 0.087)	<.001
Receipt of Economic Impact Payment in last 7 d by anyone in household	0.004 (–0.003 to 0.011)	.25
Health insurance coverage		
Uninsured	0 [Reference]	NA
Public	–0.032 (–0.040 to –0.023)	<.001
Private	–0.056 (–0.064 to –0.049)	<.001

Abbreviation: NA, not applicable.

^a^ Model also includes state wave fixed effects and a constant term, weighted using household survey weights divided by number of waves with SEs clustered at the state level. The coefficients multiplied by 100 correspond to a percentage point change in household food insufficiency.

^b^ Reflects all categories available in the public use files (other categories collected but not made available in public data files because of small numbers).

## Discussion

We found that the first round of advance CTC payments in July 2021 was associated with a 26% reduction in food insufficiency in US households with children. Nearly two-thirds of families with children reported receiving an advance CTC payment, likely a considerably lower amount than ultimately will receive it. The small percentage of households without children that reported receiving a CTC payment may have had custody changes or had a child who aged out of eligibility.

### Strengths and Limitations

This analysis is strengthened by its use of a nationally representative survey conducted nearly in real time; however, the study also has important limitations. This study is observational and relies on repeated cross-sectional data, not the same households over time. Our use of an event study and difference-in-differences regression framework, with households not targeted for the CTC advance payments (households without children present) serving as a control for households that were targeted for the CTC advance payments (households with children present), provides strong internal validity with findings that comport with results of a cross-sectional approach using self-reported receipt of the advance payments. The response rate of less than 10% is low but is typical of online surveys. Also, only 1 wave of poststudy data was available at the time of analysis. Both the response rate and early look at this association represent potential issues for generalizability. Future research using additional poststudy waves and/or alternative data sources will be necessary to conclusively establish the significance and magnitude of this association. Although it has not been peer reviewed, a recent working paper indicated a substantially similar result of a 25% decline in food insufficiency among low-income households with children after the expansion of the Child Tax Credit.^28^

A challenge in studying public assistance programs and material hardship, such as food insufficiency, is that correlational studies can be misleading, showing higher rates of hardship among those enrolled in public assistance (eg, SNAP) simply because having a low income is a necessary qualification for enrollment in such programs, whereas studies using natural experiments based on benefit or eligibility changes show reductions in hardship. However, because the CTC expansion and advance payments were near universal, only phasing out at very high incomes, this policy context and our study are not affected by this potential confounding problem between eligibility and income.

## Conclusions

The CTC expansion created a child allowance for more than 90% of the children in the US. The potential to dramatically reduce child poverty and buffer families against economic hardship is substantial. Congress could sustain and strengthen the program by making changes to the CTC in ARPA permanent and restoring eligibility for immigrant children. Ensuring that families with greatest need receive these benefits and Economic Impact Payments is also of critical importance, by creating alternative pathways to receiving these advance payments for those who earn too little to be required to file tax returns.^29^ These actions, coupled with making permanent a recent boost in SNAP benefits, could take millions of low-income families out of a cycle of hunger for good.

## Notes

Supplement. **eFigure 1.** Assessment of Outcome Pre-trends for Difference-in-Differences **eTable 1.** Household Food Insufficiency by Race and Ethnicity and Presence of Children in the Household, Census Bureau Household Pulse Survey, January to August 2021 **eFigure 2.** Percentage Point Change in Prevalence of Household Food Insufficiency Following Introduction of Child Tax Credit Advance Payments, by State **eFigure 3.** Prevalence of Household Food Insufficiency After the Introduction of Child Tax Credit Advance Payments, by State **eTable 2.** Difference-in-Differences Estimates of Introduction of Child Tax Credit Advance Payments on Household Food Insufficiency **eTable 3.** Modified Poisson Regression Estimates of Introduction of Child Tax Credit Advance Payments on Household Food Insufficiency
