# Supplementary material for: Association of the Implementation of Child Tax Credit Advance Payments With Food Insufficiency in US Households
Source: JAMA Netw Open. 2022 Jan 13;5(1):e2143296. doi: 10.1001/jamanetworkopen.2021.43296 (PMC8759005; doi:10.1001/jamanetworkopen.2021.43296)
Supplement: Supplement. — eFigure 1. Assessment of Outcome Pre-trends for Difference-in-Differences eTable 1. Household Food Insufficiency by Race and Ethnicity and Presence of Children in the Household, Census Bureau Household Pulse Survey, January to August 2021 eFigure 2. Percentage Point Change in Prevalence of Household Food Insufficiency Following Introduction of Child Tax Credit Advance Payments, by State eFigure 3. Prevalence of Household Food Insufficiency After the Introduction of Child Tax Credit Advance Payments, by State eTable 2. Difference-in-Differences Estimates of Introduction of Child Tax Credit Advance Payments on Household Food Insufficiency eTable 3. Modified Poisson Regression Estimates of Introduction of Child Tax Credit Advance Payments on Household Food Insufficiency [file jamanetwopen-e2143296-s001.pdf]

Supplementary Online Content

Shafer PR, Gutiérrez KM, Ettinger de Cuba S, Bovell-Ammon A, Raifman J. Association of the implementation of Child Tax Credit advance payments with food insufficiency in US households. *JAMA Netw Open.* 2022;5(1):e2143296. doi:10.1001/jamanetworkopen.2021.43296

eFigure 1. Assessment of Outcome Pre-trends for Difference-in-Differences
eTable 1. Household Food Insufficiency by Race and Ethnicity and Presence of Children in the Household, Census Bureau Household Pulse Survey, January to August 2021
eFigure 2. Percentage Point Change in Prevalence of Household Food Insufficiency Following Introduction of Child Tax Credit Advance Payments, by State
eFigure 3. Prevalence of Household Food Insufficiency After the Introduction of Child Tax Credit Advance Payments, by State
eTable 2. Difference-in-Differences Estimates of Introduction of Child Tax Credit Advance Payments on Household Food Insufficiency
eTable 3. Modified Poisson Regression Estimates of Introduction of Child Tax Credit Advance Payments on Household Food Insufficiency

This supplementary material has been provided by the authors to give readers additional information about their work.

© 2022 Shafer PR et al. *JAMA Network Open.*

eFigure 1. Assessment of Outcome Pre-trends for Difference-in-Differences

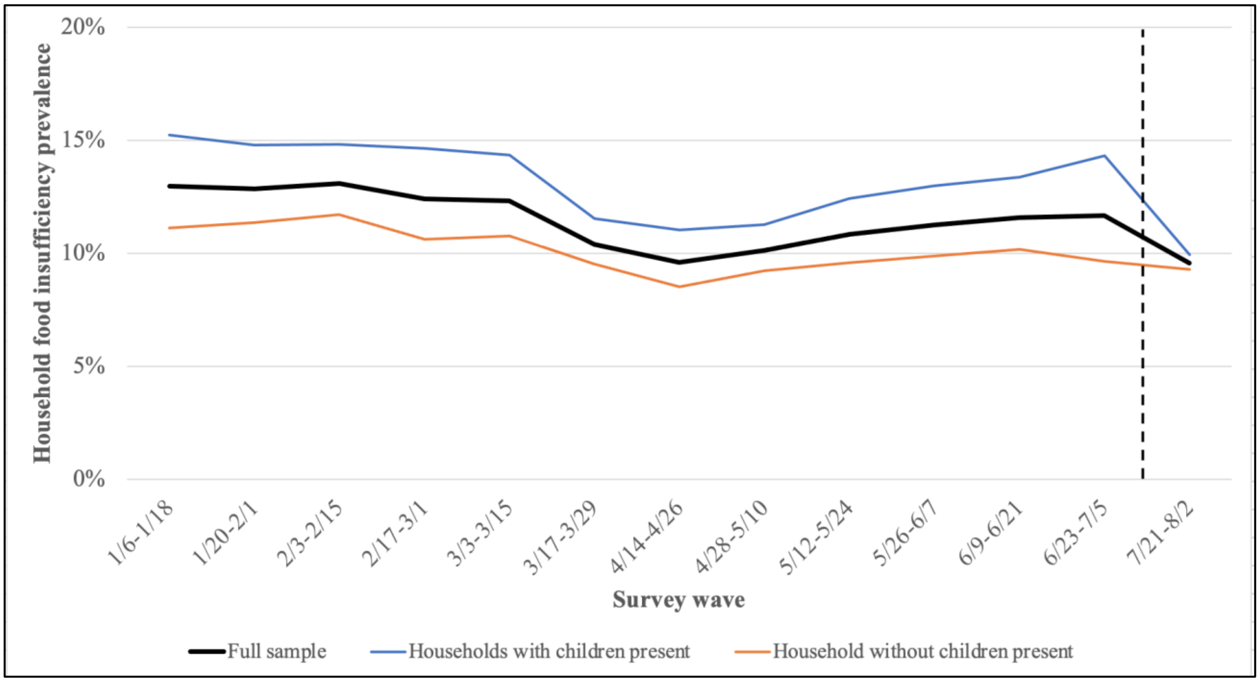

Weighted using household survey weights divided by number of waves.

Source: Authors' analysis of Household Pulse Survey data from the Census Bureau, July to August 2021 (final wave of Phase 3.2).

© 2022 Shafer PR et al. *JAMA Network Open.*

eTable 1. Household Food Insufficiency by Race and Ethnicity and Presence of Children in the Household, Census Bureau Household Pulse Survey, January to August 2021

**Sample, % (95% confidence interval)**	**Survey wave**												
	1/6-1/18	*1/20-2/1*	*2/3-2/15*	*2/17-3/1*	*3/3-3/15*	*3/17-3/29*	*4/14-4/26*	*4/28-5/10*	*5/12-5/24*	*5/26-6/7*	*6/9-6/21*	*6/23-7/5*	*7/21-8/2*
Full sample	13.0% (12.3, 13.6)	12.9% (12.2, 13.5)	13.1% (12.4, 13.8)	12.4% (11.7, 13.1)	12.3% (11.7, 13.0)	10.4% (9.8, 11.1)	9.6% (8.9, 10.2)	10.1% (9.5, 10.7)	10.8% (10.1, 11.5)	11.2% (10.6, 11.9)	11.6% (10.9, 12.3)	11.7% (11.0, 12.3)	9.6% (9.0, 10.2)
Non-Hispanic white	9.4% (8.8, 10.0)	9.0% (8.4, 9.6)	10.0% (9.3, 10.7)	9.0% (8.3, 9.7)	8.6% (7.9, 9.3)	7.6% (6.9, 8.2)	6.7% (6.1, 7.2)	7.1% (6.5, 7.6)	7.9% (7.3, 8.5)	8.4% (7.7, 9.1)	8.2% (7.6, 8.9)	8.4% (7.7, 9.0)	7.1% (6.5, 7.7)
Hispanic	20.3% (18.1, 22.5)	20.2% (18.0, 22.4)	18.6% (16.5, 20.7)	18.2% (16.2, 20.3)	19.9% (17.5, 22.3)	16.4% (14.2, 18.5)	16.5% (14.3, 18.8)	16.2% (14.1, 18.3)	17.3% (14.5, 20.1)	17.3% (15.1, 19.5	17.0% (14.6, 19.3)	18.4% (16.2, 20.7)	12.0% (10.2, 13.9)

© 2022 Shafer PR et al. *JAMA Network Open.*

Non-Hispanic Black	23.0% (20.5, 25.6)	22.1% (19.7, 24.6)	22.9% (20.2, 25.7)	23.1% (20.6, 25.7)	22.2% (19.8, 24.7)	18.4% (15.9, 20.9)	16.2% (13.7, 18.6)	19.3% (16.8, 21.8)	18.4% (16.0, 20.8)	17.1% (14.7, 19.5)	21.0% (18.4, 23.7)	20.1% (17.8, 22.4)	19.1% (16.4, 21.8)
Non-Hispanic Asian	5.7% (4.1, 7.3)	8.0% (6.1, 9.9)	6.2% (4.4, 8.0)	6.2% (4.4, 7.9)	6.3% (4.1, 8.5)	5.0% (3.4, 6.7)	4.6% (2.8, 6.5)	5.0% (3.4, 6.6)	6.4% (3.7, 9.2)	6.4% (4.7, 8.2)	5.3% (3.7, 7.0)	4.8% (3.4, 6.3)	4.9% 2.7, 7.0)
All others	18.6% (15.1, 22.1)	19.4% (16.1, 22.7)	16.6% (13.5, 19.7)	19.1% (16.0, 22.3)	17.6% (14.4, 20.8)	15.0% (11.9, 18.0)	15.2% (12.2, 18.2)	13.6% (11.0, 16.2)	13.2% (10.5, 15.9)	18.8% (15.5, 22.0)	19.4% (16.1, 22.7)	19.0% (15.7, 22.4)	15.1% (11.9, 18.3)
Households with children	15.2% (14.2, 16.3)	14.8% (13.8, 15.8)	14.8% (13.7, 15.9)	14.6% (13.6, 15.7)	14.3% (13.3, 15.4)	11.5% (10.5, 12.6)	11.0% (10.0, 12.1)	11.3% (10.3, 12.2)	12.4% (11.2, 13.6)	13.0% (11.9, 14.1)	13.4% (12.3, 14.5)	14.3% (13.2, 15.4)	9.9% (9.0, 10.9)
Non-Hispanic white	10.4% (9.4, 11.3)	8.9% (8.0, 9.9)	10.4% (9.2, 11.6)	9.0% (7.9, 10.0)	9.3% (8.2, 10.4)	7.2% (6.3, 8.2)	6.9% (5.9, 8.0)	7.0% (6.1, 7.9)	7.9% (6.9, 8.8)	8.7% (7.6, 9.8)	9.0% (8.0, 10.1)	10.0% (8.7, 11.2)	6.5% (5.7, 7.4)

© 2022 Shafer PR et al. *JAMA Network Open.*

Hispanic	22.4% (19.3, 25.6)	24.0% (20.8, 27.2)	21.0% (17.9, 24.1)	22.5% (19.3, 25.6)	21.5% (18.5, 24.5)	18.9% (15.5, 22.2)	18.9% (15.5, 22.3)	16.9% (14.0, 19.7)	19.3% (15.1, 23.5)	18.7% (15.6, 21.8)	20.3% (16.8, 23.8)	22.3% (18.9, 25.8)	12.3% (9.6, 15.0)
Non-Hispanic Black	26.0% (22.3, 29.7)	25.8% (22.2, 29.3)	25.9% (22.2, 29.5)	28.2% (24.4, 32.0)	27.0% (23.4, 30.6)	20.2% (16.8, 23.6)	18.1% (14.8, 21.4)	22.0% (18.3, 25.6)	24.3% (20.6, 28.0)	21.4% (17.9, 24.9)	21.2% (17.9, 24.5)	22.3% (19.0, 25.6)	21.1% (17.2, 25.0)
Non-Hispanic Asian	7.5% (4.5, 10.4)	8.0% (5.3, 10.6)	6.0% (3.5, 8.5)	5.8% (3.6, 8.1)	4.9% (2.5, 7.2)	6.1% (3.2, 8.9)	4.9% (2.2, 7.7)	5.7% (2.9, 8.5)	5.2% (2.6, 7.7)	5.9% (3.7, 8.2)	5.7% (3.0, 8.3)	6.0% (3.6, 8.4)	3.5% (1.9, 5.0)
All others	20.3% (14.8, 25.8)	20.7% (15.6, 25.7)	18.3% (13.5, 23.2)	21.1% (16.7, 25.5)	15.8% (11.6, 20.0)	16.6% (11.1, 22.1)	16.0% (11.4, 20.6)	14.8% (10.9, 18.7)	12.1% (8.1, 16.1)	21.3% (16.0, 26.5)	22.3% (16.8, 27.7)	20.5% (15.6, 25.4)	16.6% (11.3, 21.9)
Households without children	11.1% (10.3, 11.9)	11.3% (10.5, 12.2)	11.7% (10.8, 12.6)	10.6% (9.8, 11.4)	10.8% (9.9, 11.6)	9.5% (8.7, 10.3)	8.5% (7.8, 9.3)	9.2% (8.5, 10.0)	9.6% (8.8, 10.4)	9.9% (9.1, 10.7)	10.2% (9.3, 11.0)	9.6% (8.9, 10.4)	9.3% (8.5, 10.1)

© 2022 Shafer PR et al. *JAMA Network Open.*

Non-Hispanic white	8.8% (8.0, 9.6)	9.0% (8.2, 9.9)	9.8% (8.8, 10.7)	9.1% (8.1, 10.0)	8.1% (7.3, 8.9)	7.8% (6.9, 8.7)	6.5% (5.8, 7.2)	7.1% (6.4, 7.8)	8.0% (7.2, 8.8)	8.2% (7.3, 9.0)	7.7% (7.0, 8.5)	7.3% (6.6, 8.0)	7.4% (6.6, 8.2)
Hispanic	17.5% (14.7, 20.4)	15.9% (13.0, 18.7)	15.8% (13.1, 18.5)	13.2% (10.7, 15.6)	17.9% (14.0, 21.7)	13.7% (11.0, 16.3)	13.7% (10.9, 16.5)	15.4% (12.4, 18.5)	14.9% (11.5, 18.3)	15.6% (12.4, 18.8)	12.9% (10.0, 15.8)	14.5% (11.8, 17.2)	11.8% (9.4, 14.2)
Non-Hispanic Black	19.9% (16.2, 23.6)	18.6% (15.3, 21.9)	20.0% (15.9, 24.1)	17.5% (14.2, 20.9)	17.6% (14.2, 20.9)	16.5% (12.9, 20.1)	14.4% (10.7, 18.1)	16.5% (13.0, 20.0)	12.6% (9.8, 15.4)	12.9% (9.7, 16.1)	20.8% 16.7, 24.9)	17.8% (14.6, 20.9)	17.3% (13.6, 21.0)
Non-Hispanic Asian	4.3% (2.8, 5.7)	8.1% (5.5, 10.7)	6.3% (3.7, 8.8)	6.5% (3.8, 9.1)	7.8% (4.1, 11.4)	4.1% (2.3, 5.8)	4.4% (1.9, 6.8)	4.3% (2.6, 5.9)	7.5% (3.0, 12.0)	6.8% (4.3, 9.3)	5.0% (3.0, 7.0)	3.8% (2.2, 5.4)	6.0% (2.3, 9.8)
All others	17.1% (12.8, 21.5)	18.3% (13.9, 22.6)	15.2% (11.3, 19.1)	17.3% (12.8, 21.7)	19.1% (14.4, 23.7)	13.7% (10.3, 17.0)	14.5% (10.5, 18.5)	12.5% (9.0, 16.0)	14.2% (10.5, 17.8)	16.3% (12.4, 20.2)	17.4% (13.4, 21.5)	17.7% (13.1, 22.2)	14.0% (10.0, 18.0)

Weighted using household survey weights divided by number of waves.

Source: Authors' analysis of Household Pulse Survey data from the Census Bureau, January to August 2021.

© 2022 Shafer PR et al. *JAMA Network Open.*

eFigure 2. Percentage Point Change in Prevalence of Household Food Insufficiency Following Introduction of Child Tax Credit Advance Payments, by State

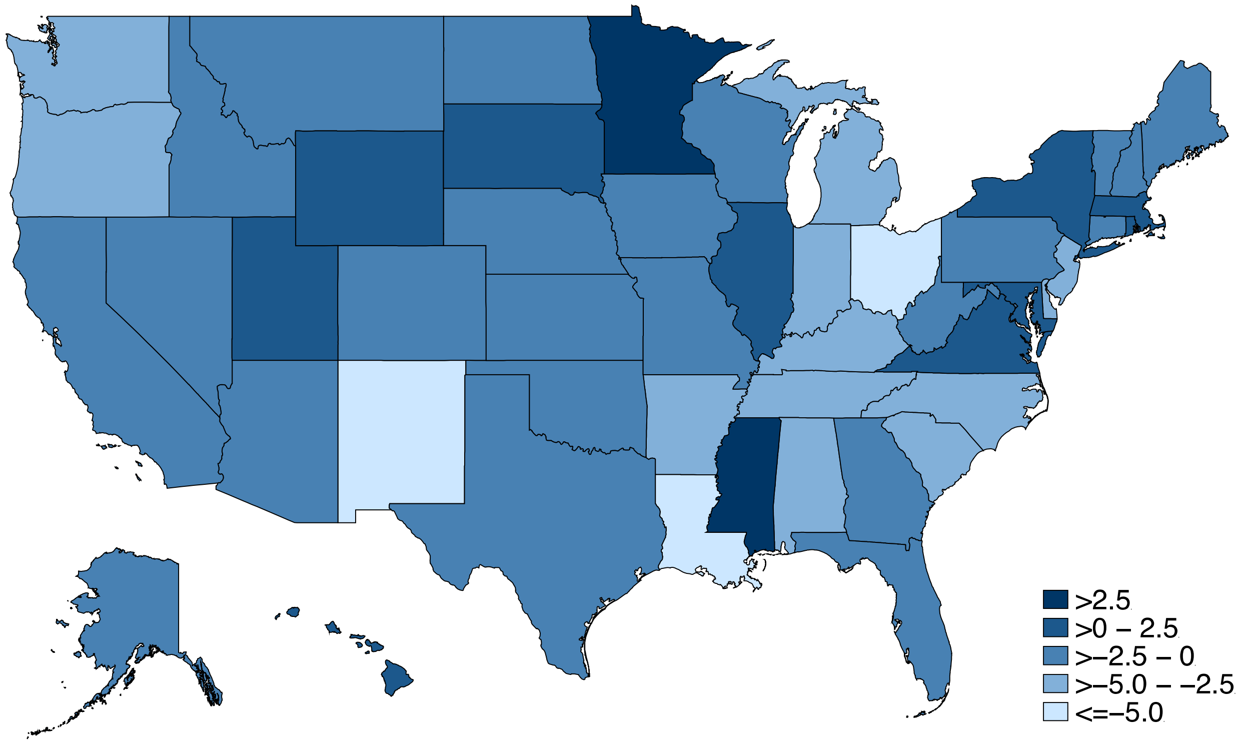

Weighted using household survey weights divided by number of waves. This captures the change in prevalence of household food insufficiency by state from the survey wave after the first advanced payment of the Child Tax Credit (July 21 to August 2, 2021) from the one right before (June 23 to July 5, 2021) [negative implies a reduction in household food insufficiency].

Source: Authors' analysis of Household Pulse Survey data from the Census Bureau, June to August 2021 (final wave of Phase 3.1 to first wave of Phase 3.2).

© 2022 Shafer PR et al. *JAMA Network Open.*

eFigure 3. Prevalence of Household Food Insufficiency After the Introduction of Child Tax Credit Advance Payments, by State

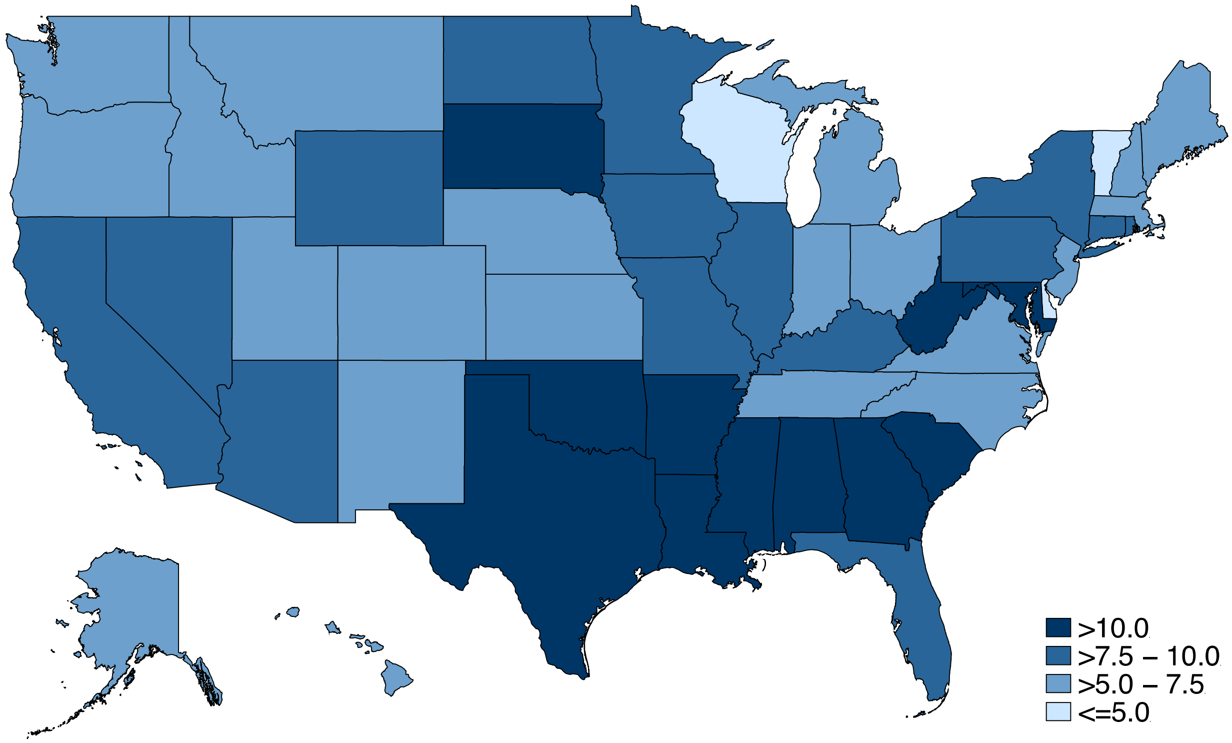

Weighted using household survey weights divided by number of waves.

Source: Authors' analysis of Household Pulse Survey data from the Census Bureau, July to August 2021 (final wave of Phase 3.2).

© 2022 Shafer PR et al. *JAMA Network Open.*

eTable 2. Difference-in-Differences Estimates of Introduction of Child Tax Credit Advance Payments on Household Food Insufficiency

**Characteristic**	**Household food insufficiency (n=585,170)**	
	Coefficient (95% confidence interval)	*P-value*
Post-period indicator	-0.025 (-0.034, -0.015)	<.001
Presence of children in household indicator	0.031 (0.023, 0.039)	<.001
Post-period *x* presence of children in household interaction	-0.022 (-0.035, -0.010)	.001
Sex at birth (%)		
Female	-0.006 (-0.009, -0.003)	.001
Male	–	–
Age group (%)		
18-24	–	–
25-44	0.051 (0.042, 0.059)	<.001

© 2022 Shafer PR et al. *JAMA Network Open.*

45-64	0.027 (0.017, 0.037)	<.001
Race/ethnicity		
Non-Hispanic white	–	–
Hispanic	0.024 (0.013, 0.035)	<.001
Non-Hispanic Black	0.053 (0.046, 0.060)	<.001
Non-Hispanic Asian	-0.009 (-0.014, -0.005)	<.001
All others	0.046 (0.036, 0.056)	<.001
Education		
Less than high school	–	–
High school or equivalent	-0.073 (-0.088, -0.058)	<.001
Some college or 2-year degree	-0.098 (-0.113, -0.083)	<.001
4-year degree or more	-0.135 (-0.149, -0.121)	<.001
Household income		
<$25,000	–	–

$25,000-34,999	-0.053<br>(-0.062, -0.043)	<.001
$35,000-49,999	-0.090<br>(-0.101, -0.079)	<.001
$50,000-74,999	-0.128<br>(-0.139, -0.118)	<.001
$75,000-149,999	-0.150<br>(-0.161, -0.139)	<.001
>=$150,000	-0.147<br>(-0.157, -0.137)	<.001
Missing	-0.129<br>(-0.139, -0.119)	<.001
Marital status		
Married	-0.025<br>(-0.029, -0.020)	<.001
Not married	–	–
Number of adults in household		
1	–	–
2	-0.015<br>(-0.020, -0.009)	<.001
3 or more	-0.005<br>(-0.014, 0.004)	.252

© 2022 Shafer PR et al. *JAMA Network Open.*

Number of children in household		
0	–	–
1	-0.014 (-0.024, -0.005)	.004
2	-0.015 (-0.025, -0.006)	.002
3 or more	–	–
Employment for respondent in the last 7 days	-0.055 (-0.060, -0.050)	<.001
Receipt of unemployment insurance benefits by respondent in the last 7 days	.002 (-0.013, 0.018)	.773
Current receipt of Supplemental Nutrition Assistance Program benefits by anyone in the household	0.022 (0.009, 0.035)	.001

© 2022 Shafer PR et al. *JAMA Network Open.*

Receipt of food aid in the last 7 days by anyone in the household	0.079 (0.071, 0.087)	<.001
Receipt of Economic Impact Payment in the last 7 days by anyone in the household	0.004 (-0.003, 0.011)	.241
Health insurance coverage		
Uninsured	–	–
Public	-0.032 (-0.040, -0.023)	<.001
Private	-0.056 (-0.064, -0.049)	<.001

Model also includes state and survey wave fixed effects and a constant term, weighted using household survey weights divided by number of waves with standard errors clustered at the state level.

Source: Authors' analysis of Household Pulse Survey data from the Census Bureau, January to August 2021.

© 2022 Shafer PR et al. *JAMA Network Open.*

eTable 3. Modified Poisson Regression Estimates of Introduction of Child Tax Credit Advance Payments on Household Food Insufficiency

**Characteristic**	**Household food insufficiency (n=585,170)**	
	Adjusted prevalence ratio (95% confidence interval)	*P-value*
Receipt of Child Tax Credit advanced payment	0.792 (0.670, 0.936)	.007
Sex at birth (%)		
Female	0.944 (0.916, 0.973)	<.001
Male	–	–
Age group (%)		
18-24	–	–
25-44	1.364 (1.277, 1.457)	<.001
45-64	1.123 (1.033, 1.221)	.007
Race/ethnicity		
Non-Hispanic white	–	–
Hispanic	1.210 (1.126, 1.230)	<.001

© 2022 Shafer PR et al. *JAMA Network Open.*

Non-Hispanic Black	1.404 (1.334, 1.477)	<.001
Non-Hispanic Asian	0.853 (0.793, 0.918)	<.001
All others	1.443 (1.350, 1.542)	<.001
Education		
Less than high school	–	–
High school or equivalent	0.787 (0.743, 0.833)	<.001
Some college or 2-year degree	0.680 (0.643, 0.719)	<.001
4-year degree or more	0.335 (0.317, 0.354)	<.001
Household income		
<$25,000	–	–
$25,000-34,999	0.885 (0.851, 0.920)	<.001
$35,000-49,999	0.747 (0.704, 0.792)	<.001
$50,000-74,999	0.519 (0.485, 0.556)	<.001

© 2022 Shafer PR et al. *JAMA Network Open.*

$75,000-149,999	0.252<br>(0.226, 0.281)	<.001
>=$150,000	0.098<br>(0.083, 0.114)	<.001
Missing	0.559<br>(0.529, 0.591)	<.001
Marital status		
Married	0.792<br>(0.763, 0.823)	<.001
Not married	–	–
Number of adults in household		
1	–	
2	0.889<br>(0.854, 0.926)	<.001
3 or more	0.987<br>(0.934, 1.042)	.618
Number of children in household		
0	–	–
1	1.119<br>(1.064, 1.178)	<.001

© 2022 Shafer PR et al. *JAMA Network Open.*

2	1.109 (1.055, 1.166)	<.001
3 or more	1.213 (1.143, 1.288)	<.001
Employment for respondent in the last 7 days	0.665 (0.645, 0.685)	<.001
Receipt of unemployment insurance benefits by respondent in the last 7 days	1.053 (0.973, 1.138)	.195
Current receipt of Supplemental Nutrition Assistance Program benefits by anyone in the household	1.025 (0.964, 1.091)	.420
Receipt of food aid in the last 7 days by anyone in the household	1.447 (1.394, 1.502)	<.001

© 2022 Shafer PR et al. *JAMA Network Open.*

Receipt of Economic Impact Payment in the last 7 days by anyone in the household	1.064<br>(1.001, 1.124)	.029
Health insurance coverage		
Uninsured	–	–
Public	0.845<br>(0.806, 0.885)	<.001
Private	0.696<br>(0.659, 0.734)	<.001

Model also includes state and survey wave fixed effects and a constant term, weighted using household survey weights divided by number of waves with standard errors clustered at the state level.

Source: Authors' analysis of Household Pulse Survey data from the Census Bureau, January to August 2021.

© 2022 Shafer PR et al. *JAMA Network Open.*
